# Transurethral resection of a bladder trigone leiomyoma: a rare case report

**DOI:** 10.1186/s12894-020-00722-2

**Published:** 2020-10-07

**Authors:** Athanasios Zachariou, Maria Filiponi, Fotios Dimitriadis, Aris Kaltsas, Nikolaos Sofikitis

**Affiliations:** 1 Urology Department, Medical School, University of Ioannina, 3 Spyridi Street, 38221 Vólos, Greece; 2 1st Urology Department, Medical School, Aristotle University of Thessaloniki, Thessaloníki, Greece

**Keywords:** Bladder, Trigone, Leiomyoma, Obstruction, Transurethral resection

## Abstract

**Background:**

Bladder leiomyomas are rare and benign tumors of the bladder. They account for 0.43% of all bladder tumors, and only 250 cases have been reported in English literature. Based on the size and localization of the lesion, their symptoms vary considerably. Women seem to be more affected, and obstructive symptoms predominate. Surgical treatment is almost always highly effective, leaving a low recurrence rate.

**Case presentation:**

We present a clinical case of a 52-year old man with macroscopic hematuria and obstructive lower urinary tract symptoms due to a large bladder trigone leiomyoma. CT and MRI showed a well-defined large bladder leiomyoma and cystoscopy established the initial findings. The patient underwent successful transurethral resection of the lesion, and pathology findings confirmed the diagnosis.

**Conclusions:**

This case report demonstrates that transurethral resection of a large bladder trigone leiomyoma is a feasible and successful procedure. Long term follow-up proves that there is neither scarring distortion of the bladder trigone area nor damage in the ureteral orifices, even though there was a thorough removal of the trigone wall.

## Background

Leiomyoma describes a benign growth of smooth muscle tissue. These tumors can occur anywhere in the body where smooth muscle is found, but are frequently located in the uterus (commonly called fibroids) or the gastrointestinal tract. Bladder leiomyoma is a rare, benign tumor with an incidence rate of about 0.43% among all types of bladder tumors [1]. Approximately 250 cases have been reported to date to English literature, including patients who had leiomyoma in a urethral location [2]. Some of the leiomyomas observed in the bladder are diagnosed accidentally, and these patients could have a variety of clinical presentations such as obstructive symptoms from the lower urinary tract, irritative symptoms, hematuria, and dysuria. Surgery is the standard treatment, and the surgical approach depends on tumor size and location at the bladder wall. Small and easily accessible tumors can be treated with transurethral resection of the bladder tumor, while an unfavorable location may require segmental resection or laparoscopic partial cystectomy [3].

Here we report a case of a large bladder trigone leiomyoma in a 52-year-old Caucasian male who presented with complaints of lower urinary tract obstruction, sporadic hematuria, and dysuria. To our knowledge, this case is one of the few reported cases of bladder trigone leiomyoma mimicking the median prostate lobe and distorting the left ureteric orifice. Our primary concern was that scar tissue following the procedure could further change the bladder trigone area, thus exacerbating patient's dysuria.

## Case presentation

A 52-year-old man presented at our clinic with gross macroscopic hematuria, and a 12-month history of the lower urinary tract symptoms. He reported frequency, urgency, nocturia, straining at voiding, dribbling at the end of urination and inability to empty the bladder. He had diagnosed with a urinary tract infection 3 months earlier. Although he had received antibiotic therapy, his urinary complaints persisted. As prescribed by a General Practitioner, his treatment was tamsulosin 0.4 mg/daily, for 2 months with limited success for the reported symptoms.

During the initial evaluation, no abnormalities were found on physical examination or in routine laboratory studies. Ultrasound of the lower urinary tract revealed a smooth endovesical bladder lesion in the bladder trigone area which mimicked the median prostate lobe as well as mild prostate hyperplasia (prostate volume 43 cc). The tumor presented peripheral hyperechogenicity. There was also mild hydronephrosis in the left kidney with no indication of kidney stone.

**Fig. 1:**
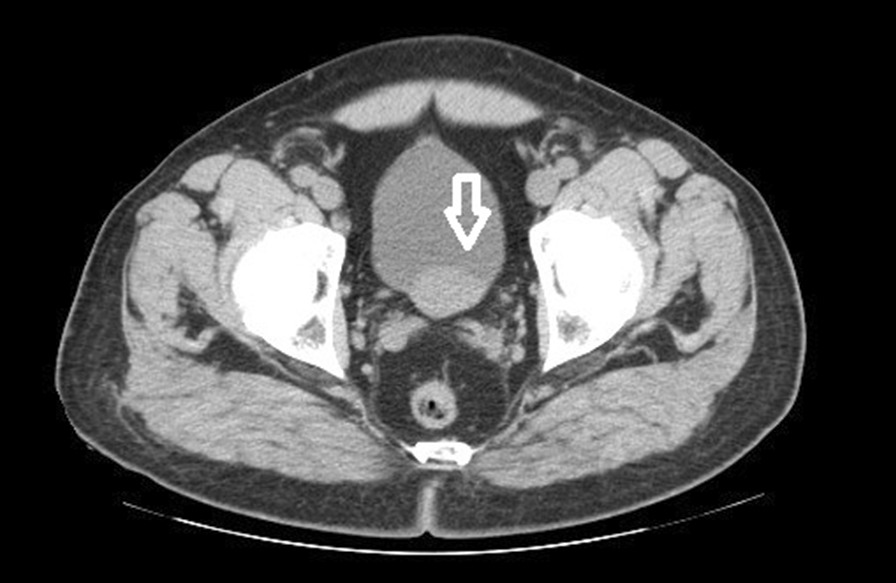
CT confirmed the presence of a solid, well-delineated mass measuring 41 × 24 mm that appeared to arise from the bladder trigone wall

**Fig. 2:**
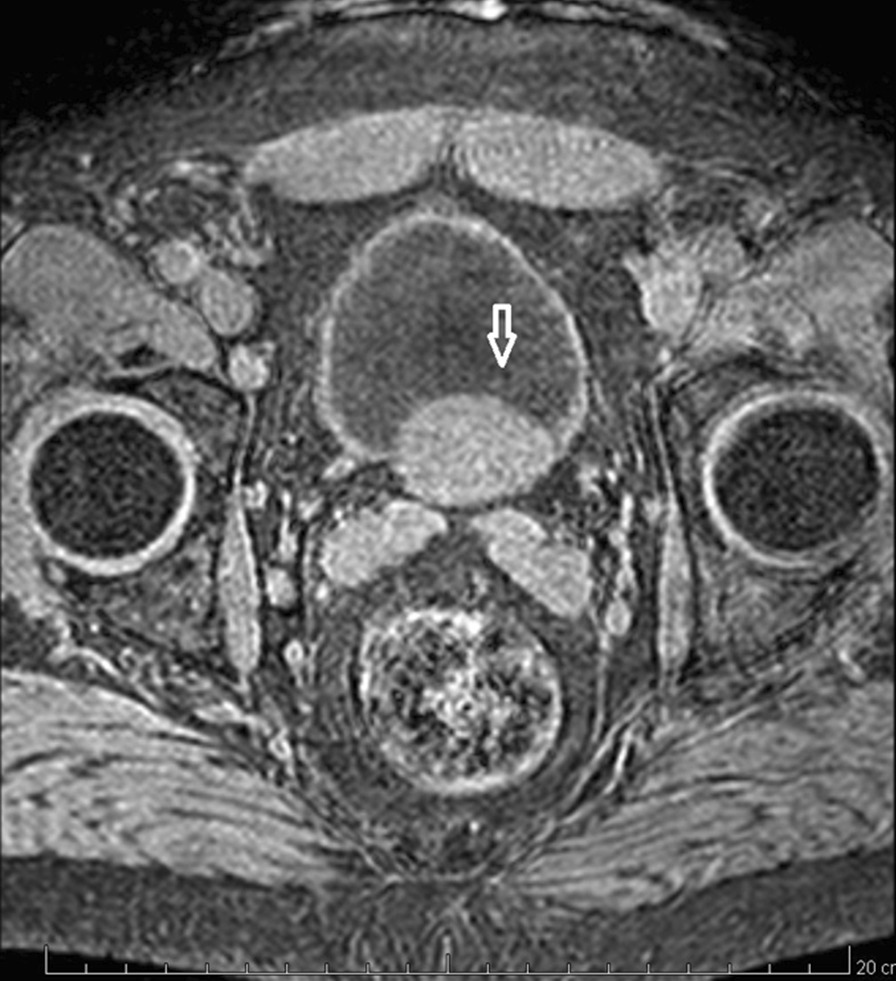
MRI revealed a well-defined 41 × 24 mm intramural mass

**Fig. 3:**
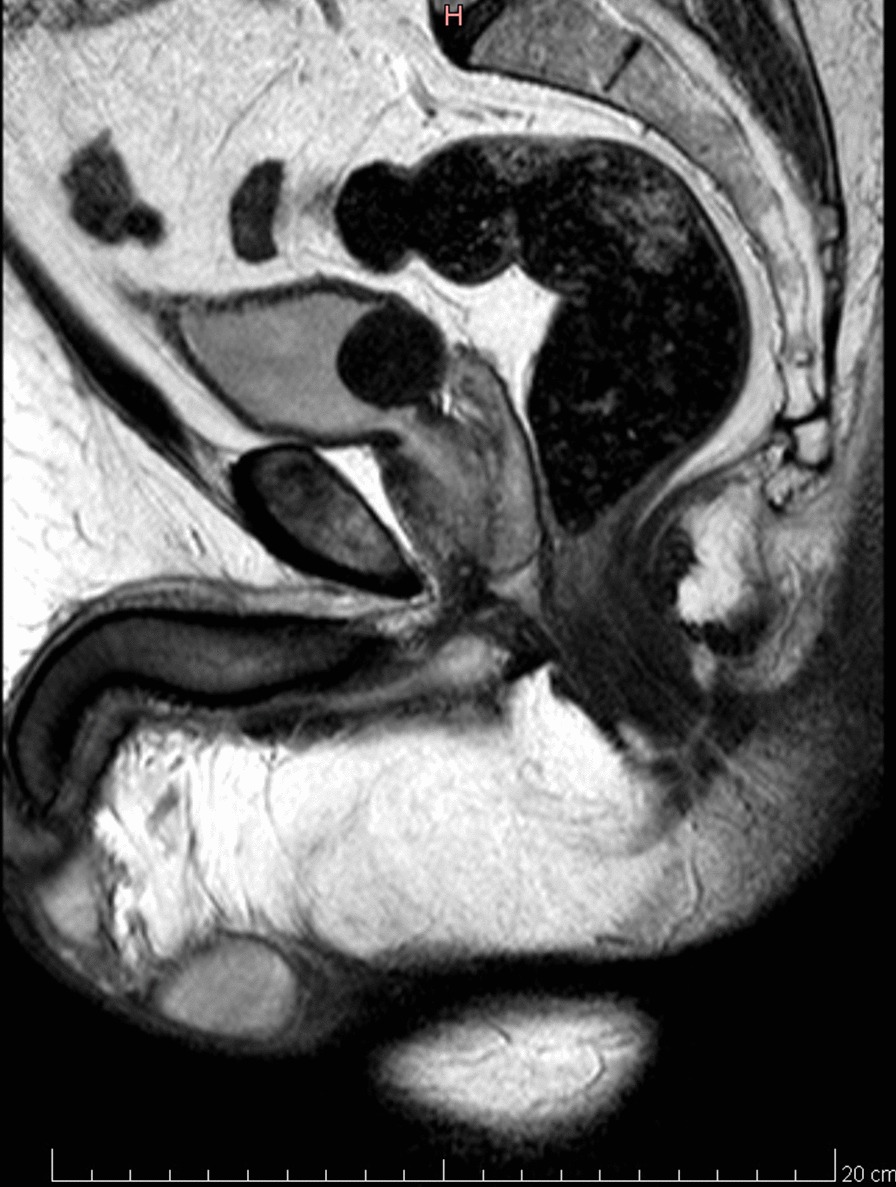
MRI sagittal view of leiomyoma

Given that asymptomatic macroscopic hematuria requires extensive investigation and the questions concerning the existence of a prostate median lobe, the patient was scheduled for computed tomography (CT). CT confirmed the presence of a solid, well-delineated mass measuring 41 × 24 mm that appeared to arise from the bladder trigone wall, independent from prostate, without any evidence of distant metastasis (Fig. 1). There was an obstructed and mildly dilated left pelvicalyceal system due to the bladder tumor displacing the left ureteric orifice. Urine cytology showed unremarkable squamous and urothelial cells and was negative for malignancy. MRI revealed a well-defined intramural mass (Figs. 2, 3).

**Fig. 4:**
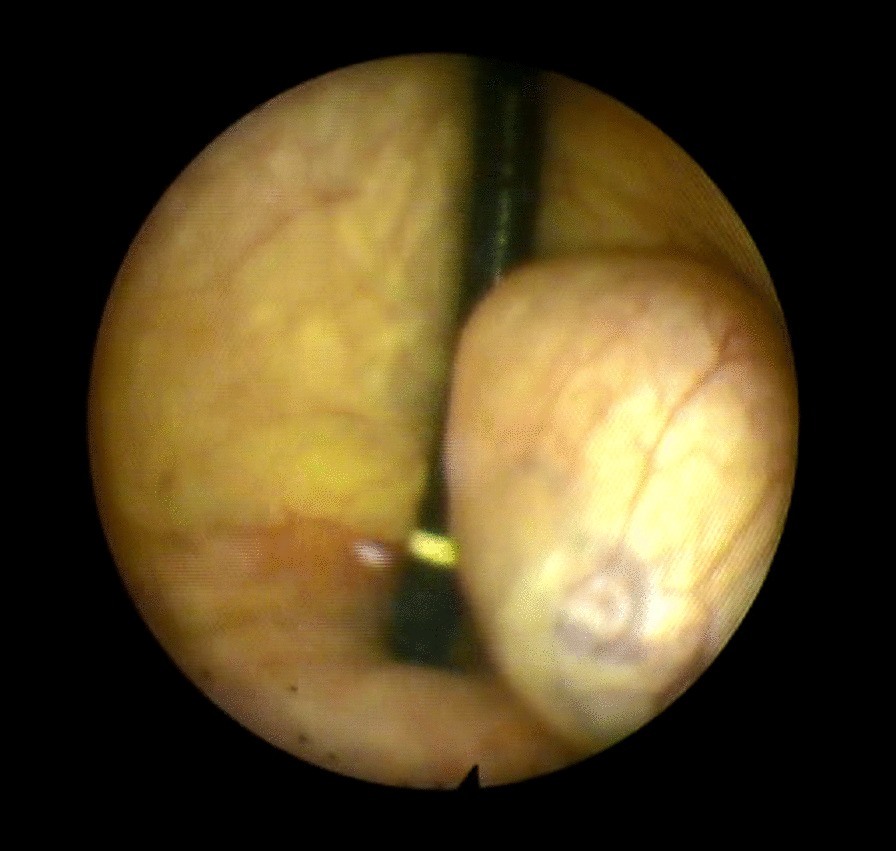
Appearance of bladder trigone leiomyoma in cystoscopy

The patient underwent cystoscopy, which revealed a protruding mass in bladder trigone next to bladder neck with normal covering urothelium. There was a distortion in the area of the left ureteric orifice, which was displaced by the mass posterior to its normal position (Fig. 4).

The patient underwent an uneventful TURBT. After the resection, a yellowish well-defined adipose tissue was revealed in the region of the bladder trigone. There are specific anatomical variations of the bladder trigone; muscularis propria bundles are nearer to the suburothelium and become smaller in caliber towards the surface. Adipose tissues can be seen in between muscularis propria bundles, which may continue extravesically or extend superficially to the lamina propria. Conversely, the outer boundary of muscularis propria with perivesical soft tissues (a landmark for staging extravesical invasion) is irregular and not well defined.

Special care was taken as regards the left ureteric orifice. Both ureteric orifices were seen to be intact. There was a significant concern about the future formation of scar tissue in the trigone area that could distort normal anatomy. The catheter was removed on the second postoperative day and the patient was discharged without any problem. Pathology proved that the lesion was a benign submucosal leiomyoma. The material consisted of interlacing fascicles of spindle-shape cells. The cells displayed no pleomorphism and contained bland-looking central spindle nuclei and eosinophilic cytoplasm. Microscopic examination of the whole tissue fragments did not reveal mitotic activity or coagulative necrosis. Immunohistochemical analysis showed an extended and robust cytoplasmic expression of smooth muscle actin (SMA) stain applied to the tissue.

Six months after surgery, an ultrasound revealed no recurrence of the tumor. A 12 month ultrasound follow-up also confirmed that the patient had no remaining lesions. Since a benign condition had already been confirmed, a usual postoperative evaluation involves the ultrasound scan. However, our concerns about a probable scarring in the blabber trigone area and distortion of ureteral orifices made a postoperative cystoscopy necessary.

## Discussion and conclusions

Bladder leiomyoma is a rare, benign, mesenchymal tumor of the bladder, with an incidence rate lower than 0.5% among all types of bladder tumors. It was first described by Kretschmer et al. in 1931 [4]. During the period 2012–2017, He et al. [5] recorded data from 21 patients in 20 reports in the English literature. The incidence of bladder leiomyoma in women is twice as high as that in men. Furthermore, middle-aged patients of approximately 50 years old present the most considerable adverse symptoms among all age groups [5].

The etiology of leiomyomas is still unknown, and multiple theories try to explain their origin. Blum’s irritative theory suggests that the reason is the presence of chronic inflammatory stimuli over the bladder wall and the detrusor smooth muscle. Some speculate that leiomyoma may arise from perivascular inflammation or chromosomal alterations [1]. Piegel’s disontogenic theory claims that its origin comes from embryogenic rests of Muller and Wolffian ducts [6]. Finally, Lips-Chutz’s theory proposes that the background of leiomyoma is secondary to an endocrine disorder, with estrogen and progesterone having a primary role. This theory supports peak incidence in females when fertile, and the presence of steroidal ovarian receptors on the tumor [7].

Leiomyomas are classified according to three different locations, i.e., endovesical, intramural, and extravesical. Endovesical is the most common location, and it corresponds to 63–86% of cases, while intramural leiomyomas are present in 3–7% and extravesical in 11–30% [6, 8].

Patients with bladder leiomyomas can be asymptomatic, but the majority of them present with obstructive symptoms (49%), irritative symptoms (38%) and hematuria (11%) [6]. Larger lesions have more symptoms, although there is a report of a bladder leiomyoma smaller than 1.4 cm in diameter that caused pain and urinary retention [9]. The location of the leiomyoma mainly attributes to the phenomenon. A leiomyoma located in the bladder trigone, like our case, may cause more severe obstructive symptoms compared with a lesion located in the bladder wall.

Traditional detection methods of bladder leiomyoma include ultrasound, CT, MRI, and cystoscopy. Ultrasound findings include homogenous, smooth lesions with peripheral hyperechogenicity [10, 11]. CT imaging is used to determine the size and extent of the tumor. Solid tumors of the bladder wall, with densities of around 30 HU and bladder displacement are the typical CT findings in bladder leiomyomas. MRI imaging is better than CT for detecting the origin and distinguishing the boundary of the tumor. Leiomyomas on MRI have a medium-signal intensity on T1-WI and homogenous low signal intensity on T1-WI, with a smooth peripheral mimicking a uterine leiomyoma. After injecting gadolinium contrast medium, contrast enhancement is variable, and degenerated areas lack enhancement [12–14]. In any case, all imaging techniques can never exclude a malignancy; therefore, only histological studies can provide an accurate diagnosis.

Leiomyomas are grossly clear-bordered, thin-capsuled solid masses with yellow-colored sectional surface. Microscopically, leiomyoma is made of intersecting smooth muscle fascicles surrounding vascular structures lined with normal endothelium and arranged as bundles extending in various directions. No mitotic activity, hemorrhage or necrosis foci are observed in these lesions. Immunohistochemically, they have positive staining with smooth muscle actin (SMA), muscle-specific actin (MSA), desmin, h-caldesmon, and vimentin. They are negative with keratins and epithelial membrane antigens (EMA). Detrusor muscle invasion is the most significant factor in differential diagnosis with leiomyosarcoma [15, 16]. Lake et al. [17] described in 1981, the only case of a leiomyoma with malignant degeneration.

The treatment of leiomyomas is determined primarily by their size, anatomic location, and relationship with the bladder wall [6]. Surgery is indicated because of the potential growth capacity of these tumors [18]. Given the benign nature of these lesions, the operation must be as conservative as possible. Treatment options include transurethral resection and open surgical excision (enucleation or/with partial cystectomy) [6]. Transurethral resection is the treatment of choice for small tumours. There are few publications reporting transurethral resections for the treatment of patients with broad bladder neck leiomyomas [19–21]. There are no relevant records about postoperative scar tissue formation in the bladder trigone area after a long term follow-up.

In some exceptional cases, specific operations were developed, such as vaginal resection for bladder leiomyoma [22]. Other minimally invasive techniques, such as laparoscopic cystotomy or robotic extramucosal excision, are also suitable [23]. In our case, the patient mainly experienced obstructive symptoms due to the specific location and the size of the leiomyoma. Therefore, transurethral resection was suitable despite the large size and the anatomic position of the leiomyoma and a successful outcome was achieved.

Recurrence after surgical removal is rare, and recurrent cases have been successfully managed with repeat enucleation or transurethral resection [24]. Having in mind that there are no guidelines regarding the follow-up of these cases, the clear suggestion is to avoid exposing the patients to invasive or radiologic investigations to detect small asymptomatic recurrences postoperatively [3].

This case report demonstrates that transurethral resection of a large bladder trigone leiomyoma is a feasible, safe and effective procedure. Long term follow-up proves that there is neither scarring distortion in the bladder trigone nor damage in the ureteral orifices, even though there was a thorough removal of trigone wall.

## Data Availability

Data are available from the corresponding author on reasonable request.

## Abbreviations

CT: Computed tomography
EMA: Epithelial membrane antigens
MRI: Magnetic resonance imaging
MSA: Muscle-specific actin
SMA: Smooth muscle actin
T1-WI: T1 weighted images
T2-WI: T2 weighted images
